# A population-based outcomes study of patients with metastatic gastric cancer receiving second-line chemotherapy: A nationwide health insurance database study

**DOI:** 10.1371/journal.pone.0205853

**Published:** 2018-10-22

**Authors:** In Sil Choi, Jee Hyun Kim, Ju Hyun Lee, Koung Jin Suh, Ji Yun Lee, Ji-Won Kim, Se-Hyun Kim, Jin Won Kim, Jeong-Ok Lee, Yu Jung Kim, Soo-Mee Bang, Jong Seok Lee, Keun-Wook Lee

**Affiliations:** 1 Department of Internal Medicine, Seoul National University College of Medicine, Seoul Metropolitan Government Seoul National University Boramae Medical Center, Seoul, Republic of Korea; 2 Department of Internal Medicine, Seoul National University College of Medicine, Seoul National University Bundang Hospital, Seongnam, Republic of Korea; University of Texas MD Anderson Cancer Center, UNITED STATES

## Abstract

**Purpose:**

The survival benefit of second-line chemotherapy in patients with metastatic gastric cancer (MGC) has recently been established. We conducted a nationwide population-based outcomes study of patients with MGC receiving second-line chemotherapy to better understand real-world treatment patterns and outcomes.

**Materials and methods:**

Data were collected from the Health Insurance Review and Assessment Service database. We identified 509 newly diagnosed patients with MGC in 2010 who received second-line chemotherapy. These patients were divided into three groups for analyses: Group A comprised all patients who received second-line chemotherapy (N = 509); Group B comprised those who received fluoropyrimidine (Fp) plus platinum as first-line treatment, followed by irinotecan-based or taxane-based regimens as second-line chemotherapy (N = 284); and Group C comprised those who received Fp plus cisplatin as first-line treatment, followed by 5-fluorouracil (5-FU)/oxaliplatin, irinotecan-based, or taxane-based regimens as second-line chemotherapy (N = 184).

**Results:**

Among patients who received first-line chemotherapy, 47.2% (509/1,078) continued to receive second-line chemotherapy. The most commonly used second-line chemotherapy regimens were 5-FU/irinotecan, 5-FU/oxaliplatin, and docetaxel. The median overall survival (OS) of all 509 patients was 5.2 months. The time from the start date of first-line chemotherapy to the start date of second-line chemotherapy > 6.1 months was an independent prognostic factor for improved OS. The type of chemotherapy regimen was not a significant factor affecting OS.

**Conclusion:**

The findings provide a better understanding of second-line treatment patterns and outcomes in patients with MGC and will help guide treatment decisions in real-world clinical practice.

## Introduction

Gastric cancer (GC) is currently the fifth most commonly occurring cancer and the third leading cause of cancer-related deaths worldwide [1]. Surgical resection is a curative treatment option for localized GC; however, local and distant recurrences commonly occur. For patients with metastatic or recurrent GC, palliative chemotherapy can effectively prolong overall survival (OS) and improve the quality of life compared with best supportive care (BSC) alone [2–4]. Although fluoropyrimidine (Fp) and platinum (P)-based combination regimens are considered established first-line treatments for patients with metastatic GC (MGC) or recurrent GC, the disease of most patients receiving first-line chemotherapy eventually progress, and the patients have a median progression-free survival (PFS) of 4–7 months. More than half of the patients do not respond to first-line chemotherapy, and among responders, the duration of response is as short as several months [5–9].

For patients with progressive disease following first-line chemotherapy, we now have evidence revealing that second-line chemotherapy with docetaxel, paclitaxel, or irinotecan results in the substantial prolongation of survival compared with BSC [10–12]. More recently, in two phase III trials of ramucirumab, which is a monoclonal antibody against vascular endothelial growth factor receptor, monotherapy or combination therapy with paclitaxel significantly improved OS compared with BSC or paclitaxel alone [13,14].

However, before obtaining recent evidence that shows the benefit of second-line chemotherapy, the proportion of patients who received further therapy after failure with first-line chemotherapy varied, largely depending on the discretion of the physician. Moreover, in the absence of standard second-line chemotherapy regimens, patients were treated with various chemotherapeutic agents [15]. Ramucirumab, a new biologic agent, was not reimbursed in many countries until quite recently. Therefore, it would be helpful to investigate real-world treatment patterns and outcomes in a large population of patients to have a better understanding of these patients and to guide treatment decisions in real-world clinical practice. To the best of our knowledge, nationwide- or population-based studies of patients with MGC or recurrent GC receiving second-line chemotherapy are very limited.

We conducted this study to assess second-line treatment patterns, outcomes, and prognostic factors associated with survival outcomes in patients with MGC using a nationwide health insurance database.

## Materials and methods

### Study population

Data were collected from the Health Insurance Review and Assessment Service (HIRA) database. In Korea, all hospitals and clinics submit inpatient and outpatient claims data on spent cost covered by the National Health Insurance or Medical Aid programs to HIRA for reimbursement. Thus, the HIRA database contains information on all claims data submitted from all hospitals and clinics. From the database, we retrieved data that included an unidentifiable code representing each individual, with diagnostic codes, demographic information, a list of medical procedures that were employed for diagnosis and treatment, and prescribed medications.

The diagnosis and procedure codes from the Korean Classification of Disease, fifth edition, as well as a supplementary code of “V193,” which is given to all patients with a confirmatory cancer diagnosis, were used to identify patients who were newly diagnosed with de novo MGC (stage IV). We already described in detail the process of identifying these cases in our previous study [16].

**Fig 1:**
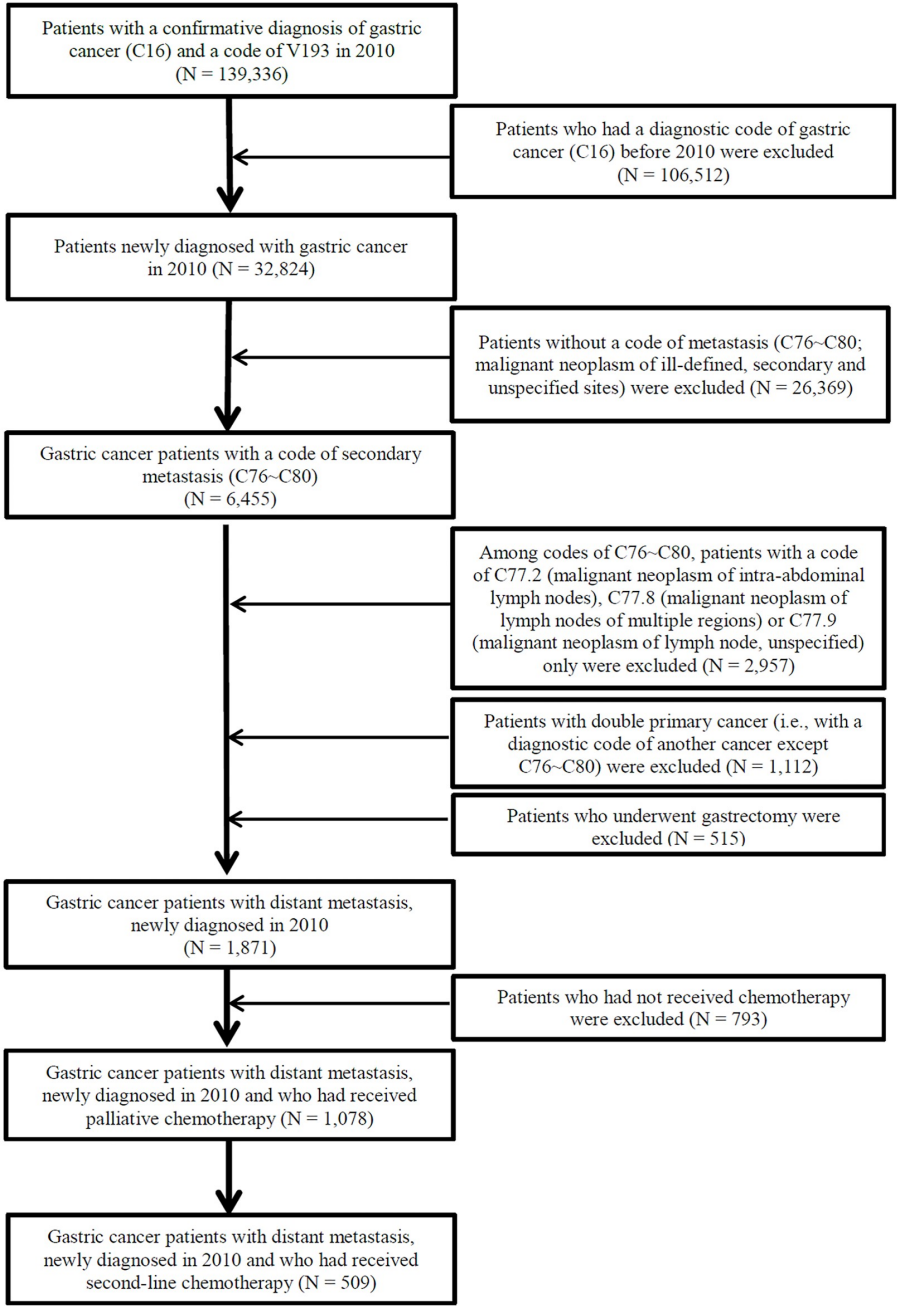
Study population and the process of case identification.

In 2010, 1,871 patients were newly diagnosed with GC with distant metastasis. Of 1,871 patients, 793 who did not receive any chemotherapy were excluded and the remaining 1,078 patients with MGC who received palliative first-line chemotherapy were identified. Among 1,078 patients, 509 who continued to receive second-line chemotherapy were finally selected as the target population of this study (Fig 1). For the selected 509 patients, the start time of second-line chemotherapy was between February 2010 and July 2013.

**Fig 2:**
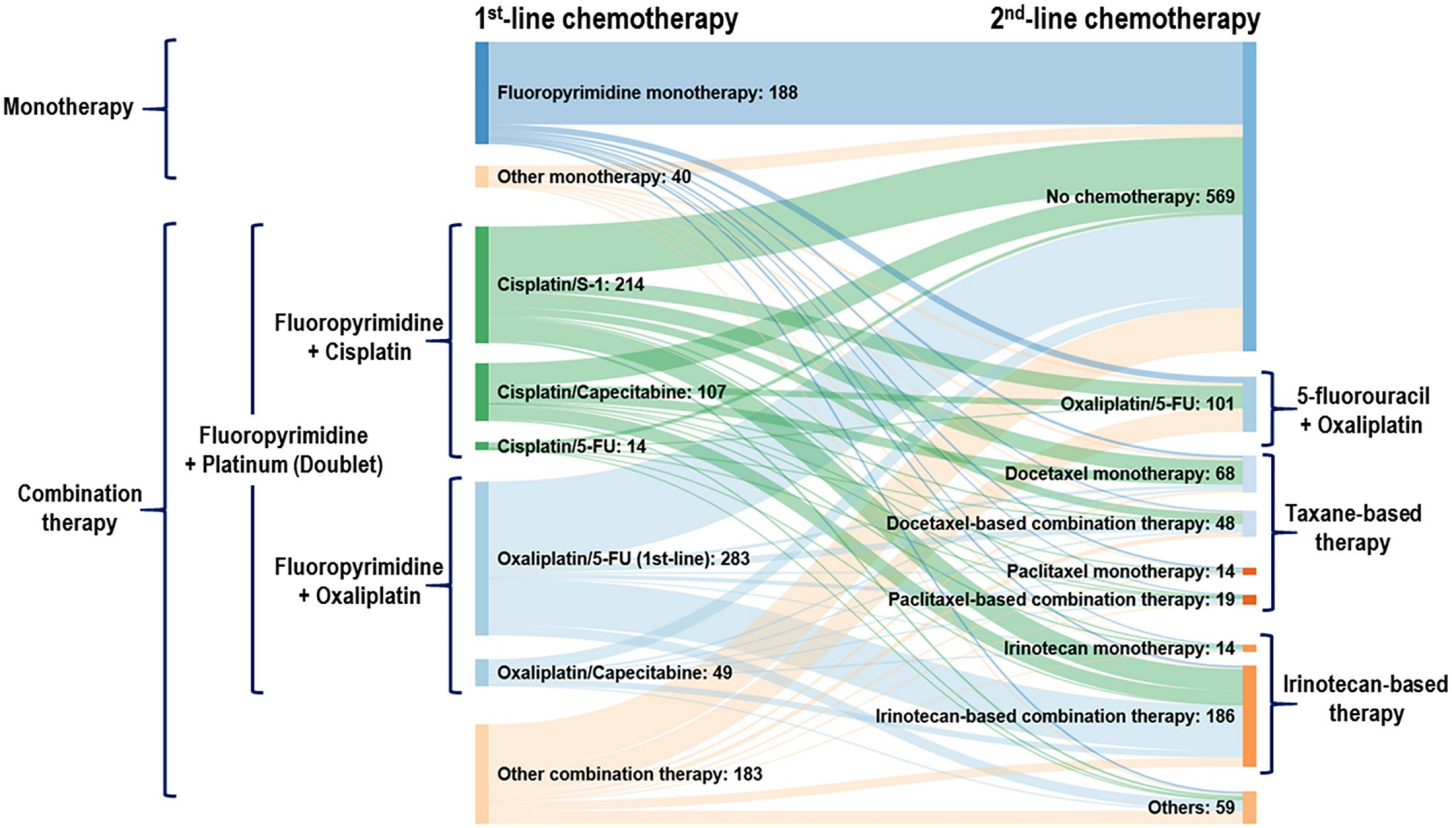
Patients’ flow from first-line to second-line chemotherapy including chemotherapy regimens (Among second-line therapy regimens, irinotecan-based combination chemotherapy (N = 186) included irinotecan plus 5-fluorouracil (N = 181), irinotecan plus cisplatin (N = 4) and irinotecan plus S-1 (N = 1); docetaxel-based combination chemotherapy (N = 48) included docetaxel plus cisplatin (N = 40) and docetaxel plus 5-fluorouracil (N = 8); paclitaxel-based combination chemotherapy (N = 19) included paclitaxel plus cisplatin (N = 12) and paclitaxel plus 5-fluorouracil (N = 7)).

The 509 patients were divided into three groups according to the treatments they received (first-line and second-line therapy): Group A comprised all patients who received second-line chemotherapy after failure with first-line chemotherapy (N = 509); Group B comprised those who received Fp plus P combination therapy as first-line treatment, followed by irinotecan-based or taxane-based regimens as second-line chemotherapy (N = 284); and Group C comprised those who received Fp plus cisplatin combination therapy as first-line treatment, followed by 5-fluorouracil (5-FU)/oxaliplatin, irinotecan-based, or taxane-based regimens as second-line chemotherapy (N = 184). The detailed patients’ flow from first-line to second-line chemotherapy including chemotherapy regimens is shown in Fig 2 using Sankey diagram (http://sankeymatic.com).

Because this study was performed using publicly released data, approval of the Institutional Review Board (IRB) was waived off (IRB number: X-1802-453-901).

### Statistical analysis

OS was estimated using the Kaplan-Meier method, and the statistical significance of differences in survival curves between the groups was assessed using a log-rank test. A prognostic model was established by searching variables that significantly influenced OS with *p* values of <0.05 in the univariable analysis. For the multivariable analysis, a Cox proportional hazards regression model was used to estimate the adjusted hazard ratio (HR) to determine the significance of specified clinical variables on OS. OS was defined as the interval from the start date of second-line chemotherapy to the date of death or last follow-up. As of October 31, 2013 (the date of data cutoff), a patient who died of any cause or who was lost to follow-up for >1 year without further information on the survival status (i.e., if the last follow-up date was before November 1, 2012) was regarded as an event case. If a patient visited a hospital or clinic at least once within 1 year before October 31, 2013 and the survival status was not reported as dead in the last claim specification, the case was regarded as a censored case. All analyses were conducted using the SAS/STAT software (version 9.4; Cary, NC, USA, SAS Institute Inc.) and IBM SPSS Statistics (version 18; Armonk, NY, USA, IBM Corp.). The statistical significance was set at *p* values of <0.05.

## Results

### Patient characteristics

In 2010, among 1,078 newly diagnosed patients with MGC who received first-line chemotherapy, 509 patients (47.2%) continued to receive second-line chemotherapy. The proportion of patients receiving second-line chemotherapy was 27.8% (76/273) among patients aged ≥70 years and 53.8% (433/805) among those aged <70 years.

The baseline patient characteristics are shown in Table 1. Group A included all patients who received second-line chemotherapy (N = 509). Of 509 patients, 85.1% of patients were aged < 70 years, with a median age of 55 (range, 25–82) years. Most patients were previously treated with Fp (88.2%) and P (82.9%), and the most commonly prescribed first-line chemotherapy regimens were 5-FU/oxaliplatin (26.5%), S-1/cisplatin (23.6%), and capecitabine/cisplatin (12.8%). For second-line chemotherapy, various regimens were prescribed; they could be classified as irinotecan-based regimens, taxane-based regimens, 5-FU/oxaliplatin, and others. Irinotecan-based regimens included irinotecan monotherapy, irinotecan/Fp, and irinotecan/cisplatin. In contrast, taxane-based regimens included taxane monotherapy (docetaxel or paclitaxel), taxane/Fp, and taxane/cisplatin. Of these regimens, the most commonly used second-line chemotherapy regimens were 5-FU/irinotecan (35.6%), 5-FU/oxaliplatin (19.8%), and docetaxel (13.4%). As mentioned in the Materials and Methods section, Group B included patients who received first-line Fp/P combination therapy followed by second-line irinotecan-based or taxane-based chemotherapy (N = 284). The most commonly used first-line chemotherapy regimens in Group B were not much different from those in Group A. However, for second-line chemotherapy, more patients received 5-FU/irinotecan (56.3%), and there were no patients who received 5-FU/oxaliplatin. Group C included patients who received Fp/cisplatin combination therapy as first-line treatment followed by 5-FU/oxaliplatin, irinotecan-based, or taxane-based regimens as second-line chemotherapy (N = 184). Most patients received S-1/cisplatin (63.0%) or capecitabine/cisplatin (33.7%) as first-line chemotherapy. As was the case in Groups A and B, 5-FU/irinotecan (35.3%) was the most commonly prescribed regimen as second-line chemotherapy, followed by docetaxel (24.5%) and 5-FU/oxaliplatin (22.3%).

**Table 1:** Patient characteristics.

Parameters	Group A (N = 509)	Group B (N = 284)	Group C (N = 184)
**Age**			
< 70 years	433 (85.1%)	255 (89.8%)	164 (89.1%)
≥ 70 years	76 (14.9%)	29 (10.2%)	20 (10.9%)
**Sex**			
Male	356 (69.9%)	198 (69.7%)	134 (72.8%)
Female	153 (30.1%)	86 (30.3%)	50 (27.2%)
**First-line chemotherapy**			
5-fluorouracil/Oxaliplatin	135 (26.5%)	115 (40.5%)	-
S-1/Cisplatin	120 (23.6%)	89 (31.3%)	116 (63.0%)
Capecitabine/Cisplatin	65 (12.8%)	50 (17.6%)	62 (33.7%)
5-fluorouracil/Irinotecan	33 (6.5%)	-	-
Docetaxel/Cisplatin	29 (5.7%)	-	-
Capecitabine/Oxaliplatin	27 (5.3%)	26 (9.2%)	-
S-1	24 (4.7%)	-	-
Others	76 (14.9%)	4 (1.4%)	6 (3.3%)
**Previously exposed drugs**			
Fluoropyrimidine	449 (88.2%)	284 (100.0%)	184 (100.0%)
Platinum	422 (82.9%)	284 (100.0%)	184 (100.0%)
Taxane	61 (12.0%)	-	-
Irinotecan	36 (7.1%)	-	-
**2**^**nd**^**-line chemotherapy regimens**			
5-fluorouracil + Irinotecan	181 (35.6%)	160 (56.3%)	65 (35.3%)
5-fluorouracil + Oxaliplatin	101 (19.8%)	-	41 (22.3%)
Docetaxel	68 (13.4%)	55 (19.4%)	45 (24.5%)
Docetaxel + Cisplatin	40 (7.9%)	28 (9.9%)	17 (9.2%)
Irinotecan	14 (2.8%)	10 (3.5%)	6 (3.3%)
Paclitaxel	14 (2.8%)	5 (1.8%)	1 (0.5%)
Paclitaxel + Cisplatin	12 (2.4%)	11 (3.9%)	1 (0.5%)
Docetaxel + 5-fluorouracil	8 (1.6%)	7 (2.5%)	3 (1.6%)
Paclitaxel + 5-fluorouracil	7 (1.4%)	5 (1.8%)	4 (2.2%)
Irinotecan + Cisplatin	4 (0.8%)	3 (1.1%)	1 (0.5%)
Others	60 (11.8%)	-	-

**Group A:** All patients who received second-line chemotherapy (N = 509)

**Group B:** Patients who received fluoropyrimidine plus platinum as first-line treatment, and then second-line chemotherapy with irinotecan-based or taxane-based regimens (N = 284)

**Group C:** Patients who received fluoropyrimidine plus cisplatin as first-line treatment, and then second-line chemotherapy with 5-fluorouracil/oxaliplatin, irinotecan-based, or taxane-based regimens (N = 184)

The median OS of all 509 patients (Group A) from the start of second-line chemotherapy was 5.2 months (95% confidence interval [CI], 4.7–5.7; S1 Fig.). Because a wide variety of regimens were prescribed as first- and second-line chemotherapy in Group A, we did not analyze and compare the survival outcomes among patients receiving particular chemotherapy regimens.

### Prognostic factors affecting survival outcomes following second-line chemotherapy (Group B, N = 284)

**Fig 3:**
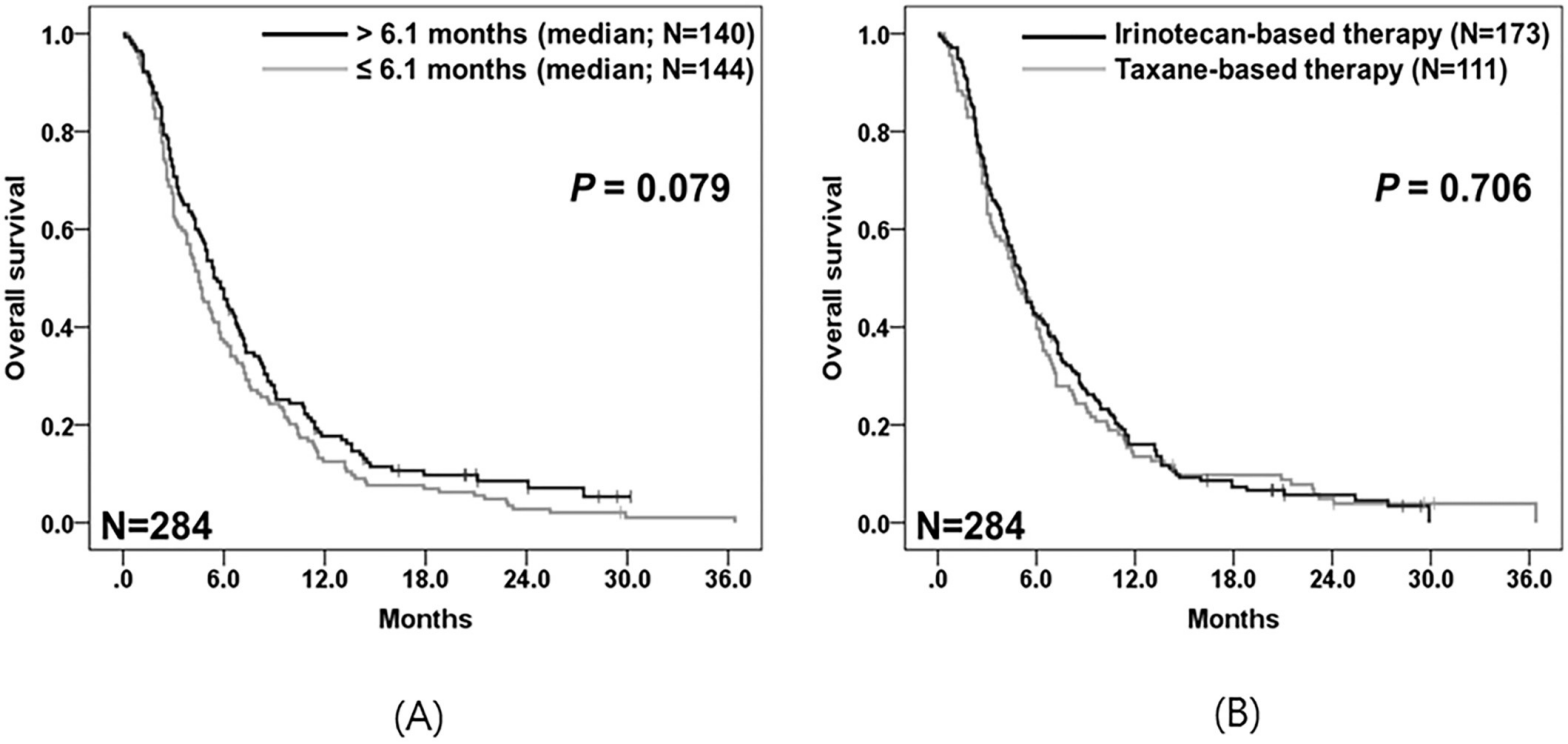
Kaplan–Meier survival curves for overall survival in Group B (N = 284). (A) Survival curves for overall survival according to the median time from the start date of first-line chemotherapy to the start date of second-line chemotherapy (TF1T2). (B) Survival curves for overall survival of patients receiving irinotecan-based (N = 173) vs. taxane-based chemotherapy (N = 111).

All patients in Group B received Fp/P combination therapy as first-line treatment, followed by second-line irinotecan-based or taxane-based chemotherapy. The univariable analyses for prognostic factors affecting OS revealed that an age of ≥ 70 years was significantly associated with shorter survival time (4.3 vs. 5.1 months; *p =* 0.048) (Table 2). The median time from the start date of first-line chemotherapy to the start date of second-line chemotherapy (TF1T2) was 6.1 months (range, 0.2–32.3 months). The median OS of patients with a TF1T2 of ≥ 6.1 months was longer than that of patients with a TF1T2 of < 6.1 months, although the difference was not statistically significant (5.4 vs. 4.5 months; *p =* 0.079) (Table 2, Fig 3A). The chemotherapy regimen was not a significant factor affecting OS (irinotecan-based vs. taxane-based regimens; 5.1 vs. 4.8 months; *p =* 0.706) (Table 2, Fig 3B). In addition, there was no significant difference in OS between patients receiving monotherapy and combination therapy (irinotecan monotherapy vs. irinotecan-based combination therapy vs. taxane monotherapy vs. taxane-based combination therapy; *p =* 0.865). However, because of a very small number of patients receiving irinotecan monotherapy, the result needs to be read with discretion (S2 Fig.). In the multivariable analyses, both age and TF1T2 were independent prognostic factors that correlated with OS following second-line chemotherapy: age of ≥ 70 years (HR 1.59, 95% CI 1.06–2.39; *p =* 0.024) and TF1T2 of ≥ 6.1 months (HR 0.77, 95% CI 0.60–0.99; *p =* 0.039).

**Table 2:** Prognostic factors related to survival outcomes in Group B (N = 284).

	N	Univariable analysis			Multivariable analysis	
		Overall survival (months; median)	*p*	Hazard ratio	95% confidence interval	*p*
Sex						
Male	198	5.0	-	1.00	-	-
Female	86	4.8	0.460	0.90	0.69–1.17	0.413
Age (year)						
< 70	255	5.1	-	1.00	-	-
≥ 70	29	4.3	0.048	1.59	1.06–2.39	0.024
Duration from first-line to second-line chemotherapy						
≤ 6.1 months	144	4.5	-	1.00	-	-
> 6.1 months	140	5.4	0.079	0.77	0.60–0.99	0.039
Chemotherapy regimens						
Irinotecan-based therapy	173	5.1	-	1.00	-	-
Taxane-based therapy	111	4.8	0.706	0.99	0.77–1.27	0.925

### Prognostic factors affecting survival outcomes following second-line chemotherapy (Group C, N = 184)

**Fig 4:**
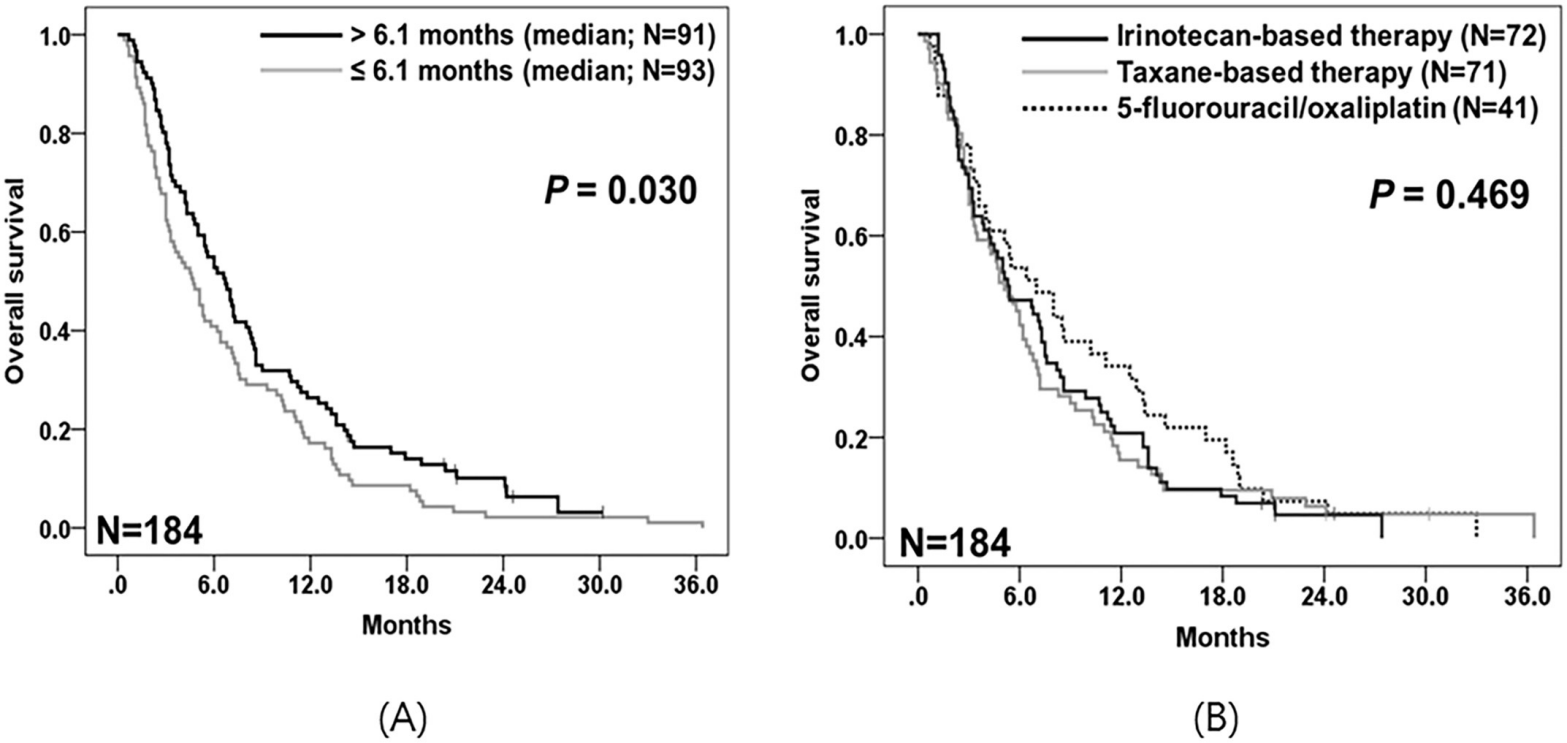
Kaplan–Meier survival curves for overall survival in Group C (N = 184). (A) Survival curves for overall survival according to the median time from the start date of first-line chemotherapy to the start date of second-line chemotherapy (TF1T2). (B) Survival curves for overall survival of patients receiving 5-fluorouracil/oxaliplatin (N = 41) vs. irinotecan-based (N = 72) vs. taxane-based chemotherapy (N = 71).

All patients in Group C received Fp/cisplatin combination therapy as first-line treatment, followed by 5-FU/oxaliplatin, irinotecan-based, or taxane-based regimens as second-line chemotherapy. In the univariable analyses for prognostic factors associated with OS, age was not a significant prognostic factor related to OS (age of ≥ 70 vs. < 70 years; 5.3 vs. 5.4 months; *p* = 0.639). TF1T2 of ≥ 6.1 months (vs. < 6.1 months) was significantly associated with improved OS (6.7 vs. 4.7 months; *p* = 0.030) (Table 3, Fig 4A). There was no significant difference in OS between patients receiving each chemotherapy regimen (5-FU/oxaliplatin vs. irinotecan-based vs. taxane-based regimens; 7.0 vs. 5.3 vs. 5.1 months; *p* = 0.469) (Table 3, Fig 4B). In the multivariable analyses, TF1T2 was the only independent prognostic factor associated with OS following second-line chemotherapy: TF1T2 of ≥ 6.1 months (HR 0.68, 95% CI 0.50–0.93; *p =* 0.015).

**Table 3:** Prognostic factors related to survival outcomes in Group C (N = 184).

	N	Univariable analysis			Multivariable analysis	
		Overall survival (months; median)	*p*	Hazard ratio	95% confidence interval	*p*
Sex						
Male	134	5.3	-	1.00	-	-
Female	50	6.2	0.295	0.84	0.60–1.18	0.324
Age (year)						
< 70	164	5.4	-	1.00	-	-
≥ 70	20	5.3	0.639	1.20	0.74–1.95	0.453
Duration from first-line to second-line chemotherapy						
≤ 6.1 months	93	4.7	-	1.00	-	-
> 6.1 months	91	6.7	0.030	0.68	0.50–0.93	0.015
Chemotherapy regimens			0.469^§^			0.268^§^
Irinotecan-based therapy	72	5.3	-	1.00	-	-
Taxane-based therapy	71	5.1	0.823^†^	0.91	0.64–1.30	0.616^†^
5-fluorouracil/oxaliplatin	41	7.0	0.259^‡^	0.72	0.48–1.08	0.110^‡^

^§^ Irinotecan-based therapy versus taxane-based therapy versus 5-fluorouracil/oxaliplatin;

^†^ Irinotecan-based therapy versus taxane-based therapy;

^‡^ Irinotecan-based therapy versus 5-fluorouracil/oxaliplatin

## Discussion

Recent evidence has demonstrated that second-line chemotherapy improves OS in patients with MGC or recurrent GC [10–14]. However, we still do not have much information regarding the subset of patients who could benefit most from second-line chemotherapy as well as chemotherapy regimens that could be accepted as standard second-line treatment. In addition, not all newly approved agents are available for clinical practice due to economic cost. In this regard, it would be helpful to investigate second-line treatment patterns, outcomes, and prognostic factors associated with survival outcomes in a large population of patients receiving treatments in real-world clinical practice.

We conducted a nationwide population-based study to assess real-world treatment patterns and outcomes in patients with MGC receiving second-line chemotherapy using a nationwide health insurance database. In 2010, of 1,078 newly diagnosed patients with MGC who received first-line chemotherapy, 509 (47.2%) continued to receive second-line chemotherapy. For second-line chemotherapy in these patients who were mostly exposed to Fp and P previously, 5-FU/irinotecan, 5-FU/oxaliplatin, and docetaxel were the most commonly prescribed regimens, and the median OS of these patients receiving second-line chemotherapy was 5.2 months. As this study was a retrospective population-based study, not prospective randomized controlled study, we were not able to compare the OS between the patients who received and those who did not receive second-line chemotherapy because it was impossible to set the start date of the OS interval in those who did not receive second-line chemotherapy. The median OS of our patient population who received second-line chemotherapy (5.2 months) was quite similar to the OS reported in previous phase III clinical trials [10–13]; in these phase III studies, second-line chemotherapy clearly resulted in the substantial prolongation of median OS (range, 4.0–5.2 months) compared with best supportive care (median OS: range, 2.4–3.8 months). In addition, we had already reported the result of an outcomes research including all patients (N = 1078) in the same patient cohort with the current study [16]. In the previous study, we showed that the OS of patients who received second-line or more chemotherapy was longer than the OS of patients who received first-line chemotherapy only, and the number of lines of chemotherapy was an independent prognostic factor for OS in all patients as well [16]. Although direct comparison of OS between patients who received and those who did not receive second-line chemotherapy is not possible in population-based outcomes research like ours, we thought the above results suggest indirect evidences of real benefit of second-line chemotherapy in patients with MGC.

In patients who received Fp/P combination therapy as first-line treatment, followed by second-line irinotecan-based or taxane-based chemotherapy (Group B), there was no significant difference in OS between patients receiving irinotecan-based or taxane-based chemotherapy. Moreover, in patients who received Fp/cisplatin combination therapy as first-line treatment, followed by second-line 5-FU/oxaliplatin, irinotecan-based, or taxane-based chemotherapy (Group C), no significant difference in OS was observed between patients receiving 5-FU/oxaliplatin, irinotecan-based, or taxane-based chemotherapy. There have been some concerns about introducing oxaliplatin after failure with cisplatin-containing regimens owing to the possibility of cross-resistance between the two agents. However, in our study, the median OS of patients receiving 5-FU/oxaliplatin as second-line chemotherapy after failure with Fp/cisplatin was numerically longer than that of patients receiving irinotecan-based or taxane-based chemotherapy. Therefore, in concordance with previous reports [17–20], oxaliplatin can be a valuable treatment option even after failure with cisplatin, and some patients with MGC or recurrent GC can benefit from 5-FU/oxaliplatin regimen in a second-line setting. In our previous study of 229 patients with MGC receiving third-line chemotherapy in the same patient cohort with the current study, the median OS of patients who received 5-FU/oxaliplatin as third-line chemotherapy was shown to be similar to that of patients who received third-line taxane or irinotecan-based chemotherapy, suggesting the benefit of 5-FU/oxaliplatin in a third-line setting even after treatment failure with cisplatin [20]. In Groups B and C, TF1T2 was a consistent independent prognostic factor affecting OS, and TF1T2 of ≥ 6.1 months (vs. < 6.1 months) was significantly associated with improved OS. However, we could not perform in-depth analysis in terms of chemo-responsiveness and tumor progression to first-line chemotherapy which must have had influences on TF1T2 because PFS or tumor response to prior chemotherapy could not be accurately assessed in this population-based retrospective study. In spite of that, TF1T2 needs to be considered and incorporated as a stratification factor when designing future clinical trials involving patients with MGC receiving second-line chemotherapy.

This retrospective study had several limitations. First, we retrieved the exact survival status from the database in only 63.9% of patients (N = 325). We assumed that patients without any claim submitted from hospitals/clinics within 1 year before the data cutoff date must have died of MGC or other causes, considering the natural course of patients with MGC who have progressive disease following palliative second-line chemotherapy. For these patients, we considered the last date of their visit to the hospitals/clinics as the date of death; therefore, the survival duration might be underestimated for some patients. However, the median OS of our patient population (5.2 months) was not much different from the OS reported in previous studies [10–13]. Second, we could not evaluate all known prognostic factors associated with survival outcomes, such as the performance status of patients, laboratory values, and tumor burdens (metastatic number and sites), because the HIRA database did not have information on these clinical variables. Furthermore, as we mentioned above, the efficacy of second-line chemotherapy in connection with PFS or tumor response to first-line chemotherapy could not be assessed by this population-based database. Third, to select MGC cases more precisely in this study, we included only MGC cases with de novo metastatic disease, excluding recurrence cases after prior curative gastrectomy, and thus, only a relatively small number of patients (509 patients) were finally selected as the target population, although this was a nationwide population-based study [16]. In addition, most patients with stage II or III disease after primary gastrectomy receive adjuvant chemotherapy with Fp- and/or P-containing regimens in Korea. Therefore, if recurrence cases after prior curative gastrectomy were included in this analysis, the results could be somewhat different.

To summarize, in this population-based study evaluating real-world treatment patterns and outcomes in patients with MGC receiving second-line chemotherapy, we observed that 47.2% of patients continued to receive second-line chemotherapy after failure with first-line chemotherapy. The median OS of these patients was 5.2 months from the start of second-line chemotherapy, and a longer TF1T2 was an independent prognostic factor for improved OS following second-line chemotherapy. The type of chemotherapy regimen was not a significant factor affecting OS. To the best of our knowledge, this is one of the few nationwide, population-based studies of patients with MGC receiving second-line chemotherapy. Overall, this study will help guide treatment decisions in real-world clinical practice and design prospective clinical trials for these patients.

## Supporting information

S1 Fig Kaplan–Meier survival curves for overall survival in the entire population (Group A, N = 509) from the start of second-line chemotherapy. (TIF)

S2 Fig Kaplan–Meier survival curves for overall survival of patients receiving monotherapy vs. combination therapy (Group B, N = 284). (TIF)
