# Supplementary figures and images for: A population-based outcomes study of patients with metastatic gastric cancer receiving second-line chemotherapy: A nationwide health insurance database study
Source: PLoS One. 2018 Oct 22;13(10):e0205853. doi: 10.1371/journal.pone.0205853 (PMC6197657; doi:10.1371/journal.pone.0205853)

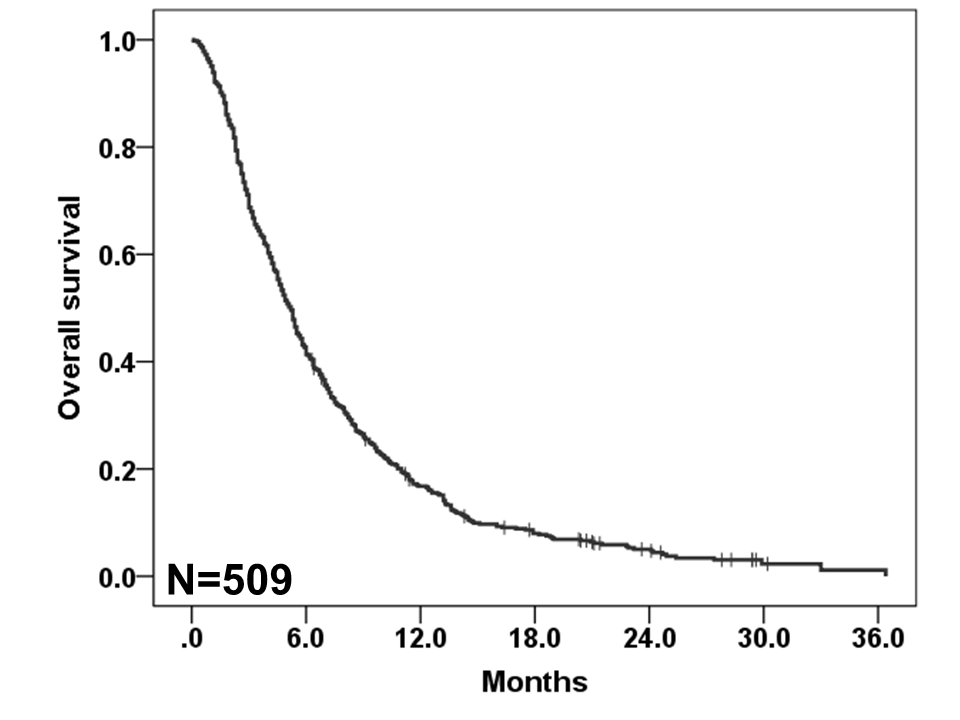

Supplement: S1 Fig — (TIF) [file pone.0205853.s001.tif]

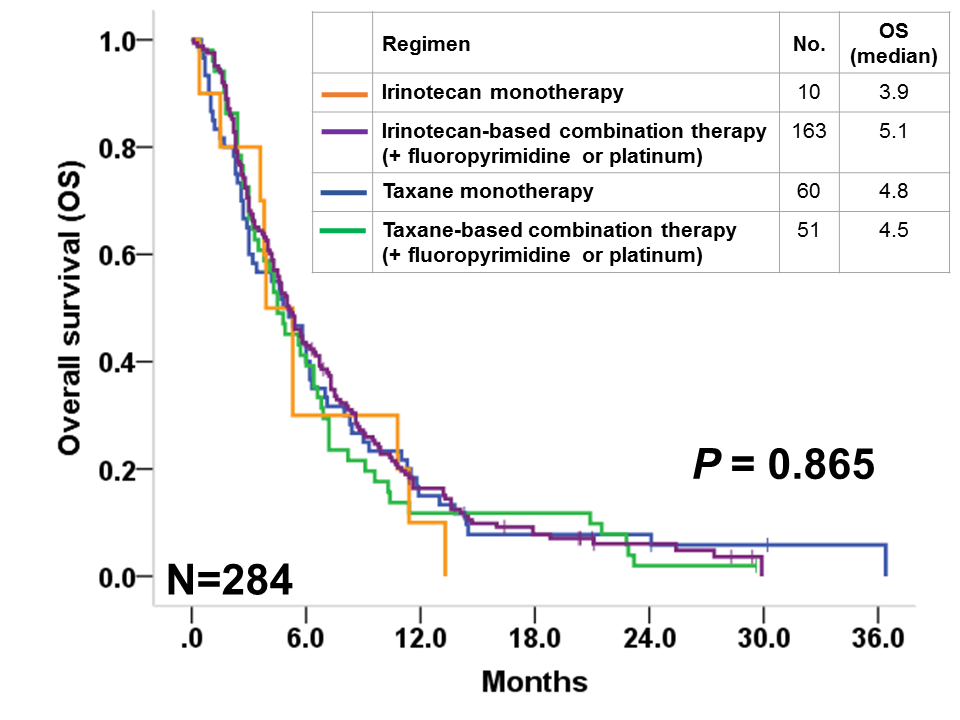

Supplement: S2 Fig — (TIF) [file pone.0205853.s002.tif]
